# Upholding the Right to Health in Contexts of Displacement: A Whole-of-Route Policy Analysis in South Africa, Kenya, Somalia, and the Democratic Republic of Congo

**DOI:** 10.3390/ijerph22071042

**Published:** 2025-06-30

**Authors:** Rebecca Walker, Jo Vearey, Ahmed Said Bile, Genèse Lobukulu Lolimo

**Affiliations:** 1 African Centre for Migration & Society (ACMS), University of the Witwatersrand, P.O. Box 76, Johannesburg 2050, South Africa; jo.vearey@wits.ac.za; 2 Global Health & Migration Unit, Department of Women’s & Children’s Health, Uppsala University, P.O. Box 256, 751 05 Uppsala, Sweden; 3 SIDRA Institute, Garowe, Puntland, Somalia; ahmed.bile@sidrainstitute.org; 4 Kinshasa School of Public Health, Kinshasa, Democratic Republic of the Congo; kennedy.lobukulu@unikin.ac.cd

**Keywords:** universal health coverage (UHC), internally displaced people (IDPs), healthcare, mental health, gender-aware, legislative frameworks South Africa, Somalia, Democratic Republic of Congo (DRC), Kenya

## Abstract

The Sustainable Development Goals commit states to Universal Health Coverage (UHC) for all; yet displaced populations—including asylum seekers, refugees, internally displaced persons (IDPs), and undocumented migrants—remain systematically excluded from national health systems across southern and eastern Africa. This paper applies a whole-of-route, rights-based framework to examine how legal status, policy implementation, and structural governance shape healthcare access for displaced populations across South Africa, Kenya, Somalia, and the Democratic Republic of Congo (DRC). Drawing on 70 key informant interviews and policy analysis conducted between 2020 and 2025, the study finds that despite formal commitments to health equity, access remains constrained by restrictive legal regimes, administrative discretion, and fragmented service delivery models. Critical gaps persist in migration-sensitive planning, gender-responsive care, and mental health integration. The findings highlight the limitations of rights-based rhetoric in the absence of legal coherence, intersectoral coordination, and political will. To realise UHC in displacement contexts, health systems must move beyond citizen-centric models and embed migration-aware, inclusive, and sustainable approaches across all stages of displacement. Without such structural reforms, displaced populations will remain at the margins of national health agendas—and the promise of health for all will remain unmet.

## 1. Introduction

### 1.1. Migration, Displacement, and Health in the SDGs

The 2030 Agenda for Sustainable Development commits all countries to achieving Universal Health Coverage (UHC) and reducing inequalities within and among countries, as articulated in Sustainable Development Goals (SDGs) 3 and 10 [1,2]. SDG target goal 3.8 specifically calls for UHC that ensures financial protection and access to quality essential services, medicines, and vaccines for all. Central to this agenda is the principle of “leaving no one behind”—a normative commitment that explicitly includes migrants, refugees, and displaced populations [3] (p. 1).

Realising the SDGs requires that migration be recognised as a cross-cutting determinant of health and development; eleven of the seventeen goals include targets and indictors directly related to migration, emphasising the importance of addressing displacement to achieve “health for all” [1,4,5,6,7]. This agenda is reinforced by key global governance instruments such as the Global Compact on Safe, Regular and Orderly Migration (GCM) [8], which operationalises SDG target 10.7, and the Global Compact on Refugees (GCR) [9], which promotes the inclusion of refugees and internally displaced persons (IDPs) in the 2030 Agenda.

However, progress remains highly uneven. As of 2024, only 16% of the SDG targets are on track to be achieved by 2030, while 84% show limited progress or reversal [10]. Marginalised populations—including undocumented migrants, refugees, asylum seekers, and IDPs—are among the most frequently excluded from policies and programmes intended to advance SDG outcomes and continue to be systematically ‘left-behind’ in efforts to achieve UHC and related health targets under the SDGs [6,10].

In this paper, we adopt the collective term ‘displaced populations’ to refer to four key groups that experience varying degrees of vulnerability and exclusion in health systems:

1. Asylum seekers: individuals seeking international protection under the 1951 Refugee Convention or regional instruments such as the 1969 OAU Refugee Convention whose claim has not yet been determined [11,12].
2. Refugees: individuals whose asylum claim has been recognised and accepted.
3. Internally displaced persons (IDPs): individuals who are those forced to flee their homes due to armed conflict, violence, human rights violations, or disasters, without crossing an international border [13].
4. Undocumented migrants: foreign-born individuals residing in a country without legal authorisation to enter, remain, or work there [14].

While these legal categories are critical for international protection and policymaking, they often fail to reflect the complex, overlapping, and fluid realities of displacement. Individuals may shift between categories, hold overlapping statuses, or fall outside formal classifications altogether [15].

### 1.2. Displacement as a Determinant of Health

Displaced populations often face a distinct and complex burden of health needs, shaped by both pre-existing vulnerabilities and conditions encountered during displacement. These include increased risk of infectious diseases (e.g., TB, HIV), sexual and reproductive health (SRH) challenges, including lack of antenatal care and exposure to gender-based violence (GBV), and intimate partner violence (IPV) [16,17,18,19,20,21,22]. These needs are compounded by poor continuity of care for chronic diseases (e.g., hypertension, diabetes), limited access to maternal and child health services, restricted access to preventive care, poor living conditions, and fear of engaging with health services due to legal insecurity [16,23,24,25].

These health risks shift over time and space and can be mapped along displacement trajectories, as people move within and between the regions, countries, and systems that impact their health and wellbeing in varied and cumulative ways [26]. Addressing the social determinants of health—such as documentation status, access to employment, safe housing, and protection from violence—is therefore integral to realising the right to health [27,28].

**Figure 1:**
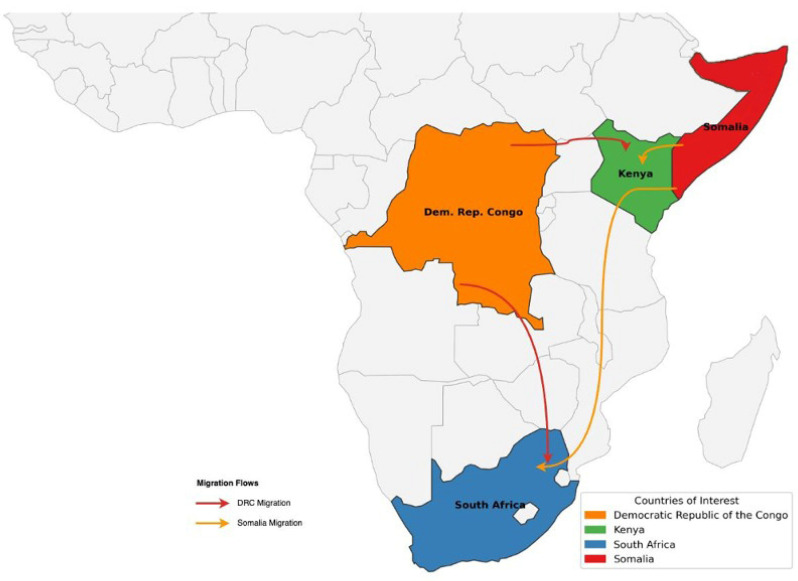
Movements of displaced people from the DRC and Somalia often follow an established migratory pathway connecting Kenya and South Africa.

This paper applies a whole-of-route approach to examine the right to health of displaced populations originating from the Democratic Republic of Congo (DRC) and Somalia—many of whom move within and beyond their countries of origin along well-established migration corridors through Kenya and South Africa (see Figure 1). Rooted in frameworks advanced by the IOM and the WHO and linked to the GCM, this approach recognises that health risks and vulnerabilities accumulate across contexts of origin, transit, and destination—shaped by shifting exposures to violence, legal precarity, and policy exclusion [29].

While the whole-of-route lens offers a valuable perspective for understanding inconsistent access to care, it is used here with awareness of scholarly critiques: namely that such approaches can be co-opted into migration management strategies that prioritise containment over rights, often reinforcing power asymmetries and undermining migrant agency [30,31]. As such, engaging this framework critically allows for an interrogation of how healthcare exclusion operates across space and time, and highlights the need to move beyond static, state-centric models of healthcare access.

Drawing on 70 key informant interviews and a review of global, continental, and regional legal and policy instruments, this paper analyses how national health systems and governance structures in the DRC, Kenya, South Africa, and Somalia—all countries with publicly funded health systems in principle—respond—or fail to respond—to the needs of displaced persons.

### 1.3. National Health Systems in Context

This section provides a brief overview of health system structures in South Africa, Kenya, Somalia, and the DRC, with attention to how decentralisation, service organisation, and governance affect access for displaced groups.

In South Africa, the health system is two-tiered, comprising a publicly funded sector and a parallel private sector. Public services are organised into three levels—primary (clinics and community health centres), secondary (district and regional hospitals), and tertiary (academic hospitals). The National Department of Health (NDoH) sets policy, which is implemented at provincial and district levels. While the public sector serves the majority, it is under-resourced and overstretched, in contrast to a well-funded private system accessible mainly to wealthier groups [32,33,34].

Kenya’s decentralised system delegates service delivery to 47 county governments, while the national Ministry of Health retains policy oversight. Health services are structured across four tiers: community (level 1), primary (levels 2–3), county referral (level 4), and national referral (levels 5–6). Despite policy commitments to UHC, displaced and mobile populations face barriers due to infrastructural disparities, administrative restrictions, and uneven implementation [35].

The DRC operates a decentralised health system overseen by the Ministry of Public Health, Hygiene, and Prevention, organised across 516 health zones and three service tiers. While national strategies like the UHC Strategic Plan (2021) indicate strong political intent, implementation is constrained by political instability, chronic underfunding, and dependency on humanitarian actors, particularly in conflict-affected zones [36].

In Somalia, the health system is fragmented and largely donor-dependent. The Federal Ministry of Health provides national guidance, while Federal Member States are responsible for service delivery. The Essential Package of Health Services (EPHS) outlines a tiered system, but implementation is limited by weak infrastructure, poor intergovernmental coordination, and insecurity. Access for IDPs remains precarious, especially outside Mogadishu and other urban centres [37,38].

### 1.4. Conceptual Framework: Legal Classification, Health Governance, and Multi-Level Policy Regimes

This paper is grounded in a conceptual framework that links legal classification, health system governance, and multi-level policy regimes to explain the systemic exclusion of displaced populations from healthcare access. It draws on a rights-based perspective, recognising health as a universal entitlement under international law, but one that is inconsistently realised in practice due to the political structuring of migration, citizenship, and state responsibility.

Access to healthcare in displacement contexts is mediated by legal status—with refugees, asylum seekers, IDPs, and undocumented migrants afforded varying degrees of recognition, rights, and entitlements. These distinctions are shaped by a hierarchy of international and regional legal instruments. Refugees and asylum seekers are protected under the 1951 Refugee Convention, its 1967 Protocol, and the 1969 OAU Refugee Convention, while IDPs are covered under the Kampala Convention. However, implementation remains fragmented, and undocumented migrants fall largely outside these protections, facing exclusion or discretionary access shaped by restrictive administrative and legal regimes [13,39].

This stratification is situated within a multi-level governance architecture that spans the global, continental, and regional levels. Globally, instruments such as the International Covenant on Economic, Social and Cultural Rights (ICESCR), the Universal Declaration of Human Rights, the Global Compact on Refugees (GCR), the Global Compact for Safe, Orderly and Regular Migration (GCM), and the WHO Global Action Plan on Refugee and Migrant Health promote inclusive access to health [8,9,40,41,42,43,44,45]. Yet these instruments are non-binding, reliant on national interpretation, and limited by political will, especially in securitised contexts [31,46,47].

At the continental level, the African Union (AU) has developed frameworks such as the AU Health Strategy (2016–2030), the Migration Policy Framework for Africa (MPFA), and the Free Movement Protocol, which articulate the principles of health equity and mobility [44,48]. At the regional level, the southern and eastern African region (SEA), including the Southern African Development Community (SADC), the East African Community (EAC), and the Intergovernmental Authority on Development (IGAD)*,* have adopted cross-border health frameworks and migrant-inclusive health strategies to promote harmonised, mobile-aware systems [33,49,50]. However, enforcement mechanisms are weak, and as this paper will show, national responses remain misaligned with these commitments.

**Figure 2:**
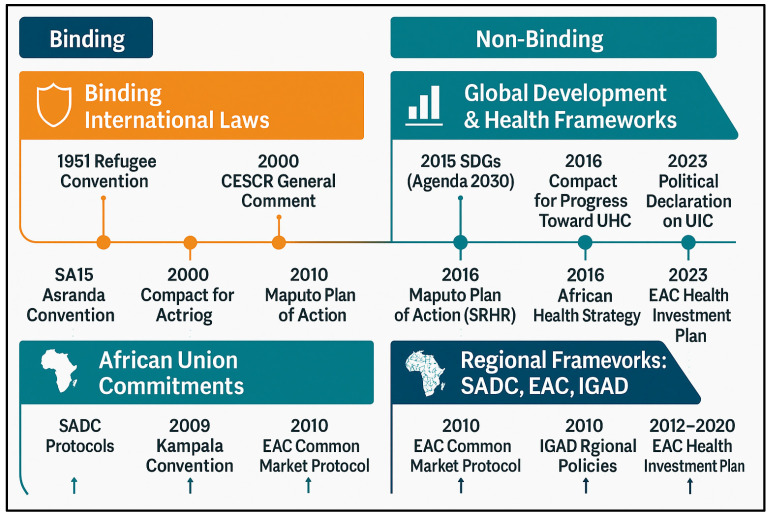
Select key migration and health frameworks: global, continental, and regional levels.

Overall, these frameworks emphasise health system harmonisation, regional health security, and inclusive access to care. Figure 2 highlights some of the key migration and health frameworks, which together form a multi-layered ecosystem of governance that supports the right to health in displacement contexts [33]. These frameworks articulate the normative foundations for the right to health and serve as policy blueprints for state-level action.

However, despite this multi-level governance architecture, health systems across sub-Saharan Africa are predominantly citizen-centric and territorially bounded, rendering mobile populations structurally invisible. Migration is rarely integrated into health system design, financing, or data collection. Implementation also remains inconsistent and often subordinated to securitised approaches to migration governance. As a result, displaced populations encounter fragmented care pathways, inconsistent entitlements, and exclusion from UHC planning. This framework positions legal classification and multi-level governance as key explanatory variables in understanding how displacement disrupts access to health and how health systems reproduce exclusion.

### 1.5. Securitisation, Systemic Exclusion, and Structural Invisibility

As health systems become entangled with immigration enforcement and border control, displaced populations are increasingly framed as risks rather than rights holders [31,51]. These dynamics legitimise restrictive policies, documentation barriers, and exclusionary practices that compound health vulnerabilities across time and space [52]. As a result, displaced persons are not only rendered invisible in national health policy, but actively excluded from essential services.

By critically examining these dynamics across four distinct national contexts, this paper reveals how structural, legal, and policy architectures shape access to healthcare for displaced populations across the SEA [33]. It exposes the disconnect between expanding normative commitments and restrictive or exclusionary implementation practices, contributing to urgent regional and global debates around UHC.

## 2. Methodology

### 2.1. Study Design

This study employed a qualitative, multi-country case study designed to examine how displaced populations experience access to healthcare across the DRC, Kenya, Somalia, and South Africa. The analysis was grounded in a whole-of-route and rights-based framework, enabling attention to the ways in which legal, institutional, and policy environments shape health access for displaced persons at different points along migratory trajectories. These four countries were selected due to their strategic importance as countries of origin, transit, and/or destination within the broader Horn of Africa and the eastern and southern African migration corridor.

### 2.2. Data Collection

Data were collected between 2020 and 2025 through two main sources: 70 semi-structured key informant interviews (KIIs) across the DRC, Somalia, Kenya, and South Africa and a comprehensive review of national and regional legal and policy frameworks in each country.

The initial phase (2020–2023), involving the co-authors and non-author contributors (listed in the Acknowledgements Section), was conducted as part of the GCRF Protracted Displacement project, ‘Improving healthcare at the intersection of gender and protracted displacement amongst Somali and Congolese refugees and IDPs (DiSoCo)’. The DiSoCo project is a GCRF-funded initiative focused on improving access to healthcare for chronic mental health conditions among Somali and Congolese populations affected by protracted displacement, conflict, and sexual and gender-based violence. It was supported by a grant (reference number ES/T004479/1) from the UK Economic and Social Research Council (ESRC) via the Global Challenges Research Fund (GCRF) development-based approaches to protracted displacement scheme. Information on the participating partner organisations can be found here: https://displacement.sps.ed.ac.uk/ (accessed on 17 June 2025). The second phase (2023–2025), included an updated review of global and regional governance frameworks as well as an updated review for South Africa, undertaken by JV and RW as part of the gendered violence and poor mental health among migrants in precarious situations Global Health Research Group (GEMMS) [33]. GEMMS is supported by the NIHR (grant ref: NIHR134629). Information on participating partner organisations can be found here: https://gemms-research.org/ (accessed on 17 June 2025).

### 2.3. Key Informant Interviews

A total of 70 semi-structured KIIs were conducted with key informants across four countries: DRC (*n* = 32), Kenya (*n* = 25), Somalia (*n* = 12), and South Africa (*n* = 25). Participants were purposively sampled to capture perspectives from stakeholders working at national and regional levels. These included government officials from health and immigration sectors, frontline healthcare providers, civil society actors, and representatives of international and local NGOs. A number of the stakeholders working for NGOs worked (or had previously worked) regionally and therefore had a strong understanding of the whole-of-route perspective. A stakeholder mapping process was used to identify relevant government and non-government organisations working at the intersection of migration and health, with additional contacts recruited through professional networks and partner organisations.

The variation in the number of participants between countries reflects the contextual access constraints, particularly in Somalia where political tensions and unrest meant it was harder to secure interviews. Access was also impacted by the COVID-19 pandemic, which coincided with the early stages of the research. During this period, health system pressures and restrictions on mobility significantly affected the availability of key informants and limited the feasibility of face-to-face interviews in several settings. While the study does not focus specifically on COVID-19, the broader effects of the pandemic on health systems and displaced populations are acknowledged and have been addressed in complementary research [53,54,55].

The interviews were structured by an interview guide developed by the South Africa team and tailored for each specific context and stakeholder group. The interview questions explored policy design and implementation processes; barriers to healthcare access for displaced populations—particularly IDPs—with attention to legal status and documentation; gender-specific challenges; and the visibility of mental health in policy and service delivery. Informed consent was obtained from all the participants. Interviews were conducted in-person or remotely (via Zoom or telephone), in English, Swahili, Lingala, and Somali. Where consent was granted, interviews were audio-recorded; in instances where participants declined, detailed notes were taken instead. All recordings or notes were subsequently transcribed and, where necessary, translated to English.

### 2.4. Policy and Legal Document Review

A comprehensive review of national and regional legal and policy frameworks was conducted, including national health laws, refugee and migration policies, IDP frameworks, regional instruments (e.g., SADC health protocols, EAC health policies, and IGAD migration frameworks), and continental and global instruments (e.g., the African Charter, Kampala Convention, GCM, GCR, and SDGs). Documents were selected based on relevance to displaced populations and their explicit or implicit reference to health entitlements or service access.

### 2.5. Data Analysis

All interviews were transcribed and coded using thematic coding in each setting by the country research team. Data were organised using NVivo (version 14) and Targeutte (2021) (in Somalia) and coded inductively and deductively, guided by the study’s conceptual framework. The codes were iteratively refined to reflect both cross-cutting themes (e.g., legal barriers, administrative discretion, and informal exclusion) and country-specific dynamics. During monthly online meetings between all the country teams over the course of twenty-four months, the coded and themes were shared, discussed, and compared. The final outputs were developed during an all-team policy analysis workshop in 2023 held at the University of the Witwatersrand, Johannesburg.

**Figure 3:**
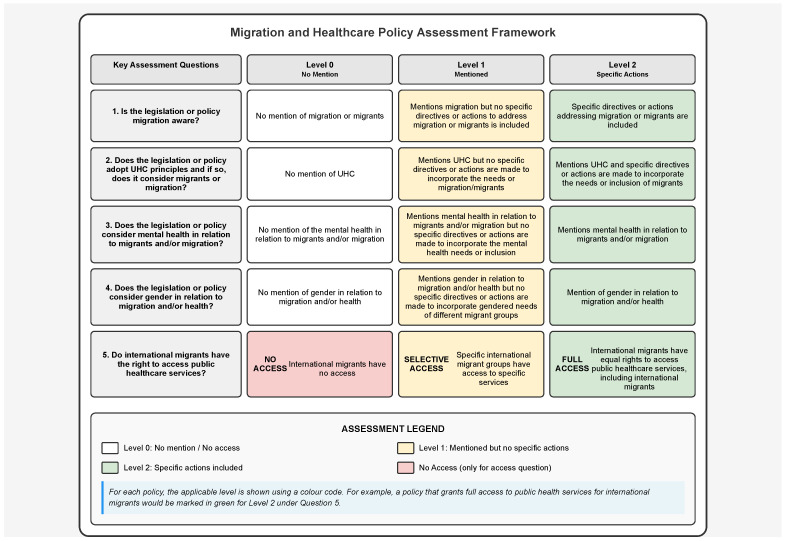
Migration and health policy assessment framework.

For each country, legislative and policy texts were systematically analysed using a structured matrix (see Figure 3) adapted from previous work undertaken by RW and JV to assess the extent to which each document addressed four intersecting concerns: (1) the right to health for migrants and displaced populations; (2) awareness of migration in healthcare planning and implementation (through access to public healthcare services); (3) awareness of mental health and psychosocial support within broader health system strategies; and (4) incorporation of gender-sensitive approaches, including protection for displaced women, girls, and other gender-diverse groups.

The documents were read through and rated against the assessment framework by 2–3 team members per country. Data was initially entered into an excel spreadsheet and then shown using the analysis framework. Global and regional governance frameworks and associated documents were identified and reviewed iteratively. Additional analysis determined the alignment of each document with key international and regional frameworks—including the SDGs, the African Union’s Migration Policy Framework, and the Kampala Convention on IDPs.

The findings were synthesised and presented in comparative tables illustrating the levels of engagement in legislation and policy with the four core focus areas for each country. These tables were adapted into graphical representations for each county and, along with the identified global and regional documents, can be found online (https://mighealth-policy-africa.org/ (accessed on 17 June 2025)). Figure 3 also highlights some of the key laws, frameworks, policies, and commitments at an international, continental, and regional level.

### 2.6. Ethical Approval

The study was conducted in accordance with the Declaration of Helsinki and approved by the Human Research Ethics Committee Non-Medical (HRECNM) of The University of the Witwatersrand, protocol code: 20-09-048 (18 September 2020). All participants provided informed consent, and interviews were conducted in line with ethical research principles, including confidentiality, voluntary participation, and the right to withdraw at any point. All participants were anonymised and all identifying information has been removed.

## 3. Results

While all four countries have made progress toward achieving UHC, none has comprehensively or consistently integrated migration, displacement, and health into national legislative and policy frameworks. Across contexts, displaced populations remain marginal in health planning, implementation, and monitoring. Where policies do exist, they are often vague, poorly resourced, and not operationalised through enforceable, coordinated strategies. This contributes to fragmented systems and entrenched exclusion, particularly for asylum seekers, refugees, undocumented migrants, and IDPs.

**Figure 4:**
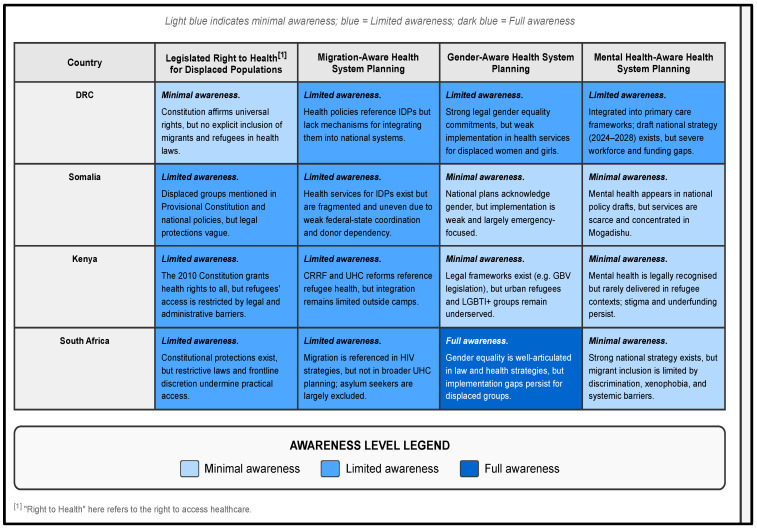
Cross-comparison of migration and health policy and planning in the DRC, Somalia, Kenya, and South Africa.

Figure 4 illustrates the variation in legal and policy engagement with the right to health for displaced populations across the four countries, including their integration into migration-aware, gender-sensitive, and mental health-responsive planning. Country-specific summaries are available online (https://mighealth-policy-africa.org/ (accessed on 17 June 2025)).

Findings from 70 key informant interviews and policy reviews are presented across five interconnected themes that expose critical implementation gaps and structural barriers to inclusive UHC.

### 3.1. Gaps Between UHC Commitments and Healthcare Access

All four countries articulate constitutional or strategic commitments to UHC; yet displaced populations—including asylum seekers, refugees, IDPs, and undocumented migrants—continue to face significant access barriers. These are shaped by legal status, administrative discretion, and structural inequities. As summarised in Table 1, the respondents described the key challenges with the implementation of UHC, exposing the limited focus on displaced populations.

Across all contexts, legal contradictions and misalignments between policy and practice persist. South Africa’s 2024 NHI Act restricts coverage for asylum seekers and undocumented migrants, despite constitutional guarantees. Kenya’s SHA and CRRF commitments reflect progressive intent, but implementation is hindered by documentation barriers and parallel systems. Somalia’s EPHS and the DRC’s UHC plan show intent but remain donor-dependent and inconsistently applied, with access for displaced persons largely ad hoc.

While constitutions affirm health rights, sectoral legislation and frontline discretion often exclude those without documentation. Respondents highlighted these disparities:

“The NHI is sending mixed signals—only some get the so-called universal access, for others, it categorises people and the most vulnerable are the most excluded”

(KII, SA 03).

“Even though refugees are entitled in theory, in practice, the system still asks for documents they cannot easily produce”

(KII, Kenya 01).

“It is impossible to implement our health plans fully because all our activities rely on funding from the donor community”

(KII, Somalia 04).

“There’s a strong will to act, but the system is overloaded. For migrants, access depends on ad hoc decisions at the facility level”

(KII, DRC 01).

These accounts illustrate how health access is stratified by legal status, place of residence (e.g., camp versus urban), and frontline discretion, leaving displaced persons systematically excluded from national health reforms.

### 3.2. Limited Migration Awareness in Health Systems Planning

Despite displacement being a major social determinant of health, the national health systems across all four countries largely overlook mobile populations in planning and service delivery. Instead, displaced communities rely on parallel humanitarian systems, fragmented coordination, and donor-driven inventions. Citizen-centric models dominate national strategies, with little cross-sectional integration or sustainable financing for migrant-inclusive health responses.

In the DRC, while IDPs are referenced in policy documents, there are no clear frameworks, financing mechanisms, or institutional mandates for their systematic inclusion. Health information systems do not disaggregate data by displacement status, and coordination with humanitarian actors remains weak [56].

In Somalia, incoherence between federal and state health authorities further fragments planning. Although IDPs form a significant share of urban populations, their access to care is almost entirely NGO-led, with minimal national oversight or integration into health sector plans.

In Kenya, the Refugees Act (2021) and CRRF participation reflect policy intent, but services for refugees remain largely parallel and dependent on external funding. As one respondent noted:

“Migrant health isn’t really planned for—it’s still left to the NGOs and project budgets.”

(KII, Kenya 03)

In South Africa, despite strong constitutional guarantees, migration-aware planning is largely absent from major reforms such as the NHI Act. Some vertical programmes (e.g., HIV, TB) acknowledge migrants, but implementation remains inconsistent and shaped by frontline discretion:

“Access depends on how you are treated at the facility, not on your rights.”

(KII, SA 02)

Across all countries, the absence of dedicated budget lines, poor inter-ministerial coordination, and lack of migration-disaggregated data inhibit the development of inclusive, responsive UHC models. While Kenya and South Africa show some policy movement, these remain constrained by securitised governance approaches. In the DRC and Somalia, displaced populations remain almost entirely within the remit of humanitarian actors. Health systems thus continue to operate in silos, leaving displaced persons structurally invisible in UHC planning.

### 3.3. Neglect of Mental Health Needs

Despite growing policy recognition, mental health remains critically underfunded, understaffed, and sidelined in UHC implementation across all four countries. Structural barriers—including stigma, legal ambiguity, and weak system integration—limit access, particularly for displaced populations.

In the DRC, the 2024–2028 draft Mental Health and Psychosocial Support Strategy is a step forward, especially in prioritising community-based care [57]. Yet with fewer than 60 trained professionals for over 90 million people, access is ad hoc and heavily reliant on short-term NGO interventions:

“There’s a strong will to act, but the system is overloaded… access depends on ad hoc decisions.”

(KII, DRC 01)

In Somalia, mental health is included in the EPHS and a draft policy, but services are almost non-existent outside Mogadishu. IDPs receive little systematic support, with temporary, donor-driven projects filling the gap:

“Mental health is a forgotten component of health services in Somalia, not only for IDPs but for even the general population.”

(KII, Somalia 11)

Kenya has a sound legal foundation in its Mental Health Policy (2015–2030) and Act (2012), but services remain peripheral, especially in refugee settings. Respondents noted underfunding and lack of integration:

“Mental health remains underfunded and understaffed.”

(KII, Kenya 02)

In South Africa, the 2023–2030 Mental Health Policy adopts a rights-based lens but fails to account for migrants [58]. Language barriers, xenophobia, and provider discretion exacerbate exclusion:

“Migrants are effectively invisible in mental health service delivery.”

(KII SA 05)

Across all settings, mental health inclusion in policy has not translated into service provision. Displaced access remains fragmented, short-term, and external to national systems—deepening health inequities and undermining UHC goals.

### 3.4. Insufficient Gender-Sensitive Health Programming

Gender-sensitive health responses across all four countries remain limited in scope and poorly implemented for displaced populations. While national policies often reference gender equity, practical measures are narrow—frequently restricted to maternal health—and fail to address sexual and reproductive health (SRH), GBV, or the needs of LGBTQ+ individuals.

In South Africa, despite progressive laws like the Equality Act (2000) and the 2019 SRHR Policy, enforcement is inconsistent. Xenophobia and administrative hurdles disproportionately affect displaced women and LGBTQ+ migrants, particularly in public health facilities.

In Kenya, the GBV Policy (2014) recognises risks faced by refugee women, but services are largely camp-based. Urban refugees and undocumented women remain underserved, and stigma hinders access: “many cases go unreported” (KII, Kenya 02).

In Somalia, gender programming is limited to maternal care in IDP camps, typically delivered by NGOs. National strategies acknowledge displaced women, but coordination is weak and coverage patchy:

“We have to prioritise essential packages such as maternal and child health.”

(KII, Somalia 02)

While the National Gender Policy (2016) and Health Sector Strategic Plan (HSSP III) acknowledge displaced women’s needs, service coverage is fragmented and poorly coordinated.

In the DRC, gender equity is constitutionally guaranteed and supported by the 2015 Law on Parity. However, displaced women’s access to SRH and GBV services remains severely constrained outside urban areas and dependent on short-term humanitarian support.

Across all countries, systemic barriers—including documentation requirements, funding gaps, and weak intersectoral collaboration—undermine access to comprehensive, gender-responsive care for displaced populations. LGBTQ+ migrants are almost entirely absent from health system planning and service delivery.

### 3.5. Structural Barriers to Inclusive Health Systems

Displaced populations face exclusion not only due to legal or policy gaps, but also because of deep-rooted structural constraints that undermine health system responsiveness. These include chronic underfunding, weak governance, reliance on parallel humanitarian structures and an overemphasis on securitised migration governance, leading to the restriction of rights.

In the DRC, instability and poor financing leave health services for displaced people heavily donor-dependent. Local clinics close when funding ends:

“When humanitarian funding stops, the clinics for displaced people often close too.”

(KII, DRC)

In Somalia, decentralisation, insecurity, and fragmented mandates compromise service delivery. IDP-hosting areas often lack basic infrastructure, and government leadership remains minimal.

In Kenya, documentation barriers tied to refugee status and SHA enrolment restrict access to care for displaced people. Disconnects between the Ministry of Health and Refugee Affairs Secretariat limit integrated planning.

In South Africa, constitutional rights are undermined by restrictive laws such as the Refugees Amendment Act and the Border Management Authority (BMA) Act. The dual public–private health system and rising xenophobia further entrench exclusion.

Across contexts, siloed governance, donor dependence, and policy incoherence prevent the development of sustainable, inclusive systems.

### 3.6. Synthesis of Findings

Overall, health access for displaced groups is shaped by a convergence of legal exclusion, political neglect, and structural fragmentation. Without migration-aware, rights-based, and system-integrated reforms, UHC will remain aspirational rather than actionable for those most at risk.

The findings from South Africa, Kenya, Somalia, and the DRC illustrate persistent and systemic gaps between national policy commitments to health equity and the lived realities of displaced populations. Despite formal recognition of the right to health and public commitments to UHC, displaced communities continue to experience structural exclusion across all stages of displacement. These findings—structured across five themes—highlight not only service-level gaps, but also the deeper governance failures, legal contradictions, and administrative practices that shape healthcare access for displaced persons.

The discussion below interprets these findings in relation to broader debates in the literature on global health, health equity, and migration governance. In doing so, it considers the political economy of exclusion, the limits of rights implementation, and the implications of the whole-of-route framework for rethinking UHC in displacement contexts.

## 4. Discussion

### 4.1. From Normative Commitment to Implementation: The Reality of Policy–Practice Gaps

While policy commitments to UHC are present in each country, their implementation remains fragmented and exclusionary. As the findings show, access for displaced populations is shaped less by normative frameworks and more by legal status, administrative discretion, and political (lack of) will.

Reflecting the well-documented implementation gap in global health—where formal commitments are undermined by weak state capacity, inconsistent enforcement, and legal contradictions—these finding highlight the need to engage more deeply with the structural and political drivers of exclusion, particularly containment-oriented migration governance [31,59]. As Storeng and Behague argue, the rhetoric of integration often masks continued structural drivers that bias the global health efforts towards narrow, vertical objectives [59].

These gaps are most evident where parallel systems of care exist. While humanitarian actors provide essential services outside national systems, ostensibly to ensure that those falling through the gaps receive care, this approach can create fragmentation and perpetuate exclusion [60]. Furthermore, without the support of national health systems and mechanisms to enforce the right to health for all residents—regardless of status—UHC becomes an empty promise.

### 4.2. Migration-Blind Systems and the Limits of National Health Planning

While exclusion from healthcare often stems from restrictive laws and securitised migration policies, as discussed above, our findings also highlight how structural and institutional blind spots within national health systems reinforce and reproduce this exclusion. Across all four countries, health planning continues to reflect static, citizen-centric models that lack inter-sectoral approaches and fail to accommodate the realities of displacement and mobility.

Rather than being integrated into core planning and budgeting frameworks, services for displaced populations are often delivered through donor-led, humanitarian channels, particularly in Somalia and the DRC. This reliance undermines national ownership and sustainability. Even in Kenya and South Africa—where policy frameworks are comparatively progressive—migrant health remains marginal to broader health system reform and is constrained by (deliberate) legal fragmentation and implementation gaps [61,62,63].

This pattern reflects wider critiques in the literature on vertical governance, short-term humanitarianism, and the limits of sectoral health reforms in contexts of mobility [59,64,65]. It also underscores the importance of a ‘whole-of-route’ approach to health systems—one that recognises mobility not simply as a linear journey, but as a continuous and shaping force in people’s lives and health needs [29]. For displaced populations, access is not only about rights at a destination, but about coherent, coordinated, and inclusive systems across all stages of movement. Without this structural integration, health systems will continue to reinforce invisibility and exclusion, undermining progress toward UHC.

### 4.3. Mental Health as a Neglected Pillar of Displacement Health

Mental health emerged as one of the most under-resourced and poorly integrated components of health systems in all four countries. Despite growing global attention to the mental health needs of displaced populations [66], services remain minimal, culturally inappropriate, and disconnected from broader UHC frameworks [17,67]. This neglect has severe consequences with the “daily stressors” faced by migrants—such as poverty, discrimination, and legal uncertainty [54,68]. These daily stressors accumulate along the displacement route, compounding trauma and underscoring the need for integrated, migrant-aware mental health services.

Yet mental health services remain siloed, often delivered by underfunded NGOs rather than embedded in primary care. In South Africa, while mental health services are extremely limited across the public health system, migrants face additional challenges accessing help, including stigma, discrimination, and exclusion, reinforcing structural discrimination [53,54]. Addressing this gap requires not just funding but a paradigm shift in how mental health is conceptualised and delivered within UHC systems [17,20,69].

### 4.4. Gender, Intersectionality, and the Reproduction of Inequality

Health systems across the four countries fail to operationalise gender- and sexuality-inclusive care for displaced persons. While policies may contain gender-sensitive language, programming often defaults to binary frameworks and fails to address the needs of LGBTIQ+ persons or survivors of sexual and gender-based violence [39,70].

This reflects a broader failure to apply intersectional approaches that acknowledge how gender, legal status, race, and class intersect to shape compounded vulnerabilities. For example, displaced women and gender-diverse individuals experience heightened exposure to violence, reproductive health risks, and stigma—yet rather than being explicitly addressed these needs are marginalised within both humanitarian and public health systems [39,71,72].

### 4.5. Civil Society’s Role and the Crisis of Sustainability

In the absence of state responsive systems, civil society has emerged as the de facto safety net. However, this role is increasingly unsustainable as chronic underfunding, political pressure, and lack of formal recognition constrain their impact and leave health access vulnerable to political shifts.

Kenya and South Africa illustrate this tension: CSOs have litigated successfully for migrant health rights, but implementation remains uneven. In Somalia and the DRC, where the state has limited capacity, humanitarian actors dominate service provision but are largely excluded from national UHC planning. This reflects the enduring humanitarian–development divide and underscores the need for genuine state ownership and long-term investment in inclusive health systems [73]. With the current US funding freeze, this is likely to get much worse [74].

### 4.6. Toward Transformative Health Governance

These findings highlight not just sectoral shortcomings but fundamental governance failures. As we have shown, the exclusion of displaced populations from UHC is not accidental—it is structured by legal, political, and economic systems that treat mobility as exceptional and threatening. As is the case with South Africa, the NHI Act and the Immigration Act undermine the facilitation of UHC through the South African Constitution and the National Health Act. By making the legal status of migrants the most significant determinant of health, the realisation of UHC is pushed further away [75]. A genuine commitment to UHC and the right to health must therefore be grounded in legal harmonisation, intersectoral collaboration, and accountable public health governance that prioritises “protection and rights over punishment and exclusion [61].

The whole-of-route, rights-based framework provides a critical lens to rethink how health systems respond to displacement. It calls for coordinated responses across origin, transit, and destination countries and insists that the right to health must apply irrespective of legal status or geographic location. Only through such structural reforms can health systems begin to uphold the promise of “health for all” in contexts of displacement.

Realising the right to health for displaced populations will require not only rhetorical inclusion but a transformation of health governance: one that is legally coherent, cross-sectoral, adequately resourced, and rooted in rights rather than risk.

## 5. Conclusions

Despite strong normative commitments to UHC across South Africa, Kenya, Somalia, and the DRC, displaced populations remain structurally excluded from national health systems. Legal classification, administrative discretion, and fragmented governance architectures consistently undermine access to care. This paper—guided by a whole-of-route, rights-based framework—demonstrates that policy intent does not translate into inclusive practice.

Overall, three key insights emerge. First, contradictions between constitutional guarantees and restrictive sectoral laws produce fragmented health entitlements. Legal status remains the dominant determinant of access, overriding universalist language. Second, rights-based frameworks are necessary but insufficient without political commitment, legal harmonisation, and intersectoral coordination. Third, a genuine shift toward inclusive health governance requires migration-aware planning and investment across displacement contexts—not just in countries of destination.

UHC for displaced populations is achievable but demands structural reforms. These include embedding migration into health financing, service design, and data systems; ensuring legal coherence between health and immigration policy; and reducing dependence on donor-led parallel systems. Without such measures, displaced populations will continue to be marginalised—and UHC will remain an unrealised promise for some of the world’s most vulnerable groups.

## Figures and Tables

**Table 1:** Comparative overview of Universal Health Coverage (UHC) approaches in DRC, Somalia, Kenya, and South Africa.

Country	UHC Strategy	Implementation Status	Attention to Displaced Populations
Democratic Republic of the Congo (DRC)	National Health Development Plan; UHC strategy	Policy intent exists; rollout is slow and aid-dependent	Very limited inclusion of displaced groups; weak, ad hoc, and inconsistent
Somalia	Essential Package of Health Services (EPHS); UHC roadmap	Fragmented implementation; limited to urban centres and IDP camps	IDPs included on paper, but service delivery is weak and inconsistent
Kenya	UHC by 2030: Social Health Insurance Act, Primary Health Care Act, Digital Health Act	Over 17.8 million enrolled in the Social Health Authority (SHA); reforms underway	Some inclusion via SHA, but registration and documentation barriers persist for refugees and informal migrants
South Africa	National Health Insurance (NHI) Act: single-payer, equity-focused model	Signed into law (2024); gradual rollout; legal and financial hurdles remain	Policies acknowledge migrants but exclude asylum seekers and undocumented migrants from comprehensive coverage

## Data Availability

The legislation, policies, and frameworks identified and utilised in this paper can be found online: https://mighealth-policy-africa.org/ (accessed on 17 June 2025).
